# Estimating the Impact of Tobacco Parity and Harm Reduction Tax Proposals Using the Experimental Tobacco Marketplace

**DOI:** 10.3390/ijerph18157835

**Published:** 2021-07-23

**Authors:** Roberta Freitas-Lemos, Diana R. Keith, Allison N. Tegge, Jeffrey S. Stein, K. Michael Cummings, Warren K. Bickel

**Affiliations:** 1 Fralin Biomedical Research Institute at Virginia Tech Carilion, Roanoke, VA 24016, USA; rflemos@vtc.vt.edu (R.F.-L.); drkeith@vtc.vt.edu (D.R.K.); ategge@vt.edu (A.N.T.); jstein1@vtc.vt.edu (J.S.S.); 2 Department of Statistics, Virginia Tech, Blacksburg, VA 24060, USA; 3 Department of Psychiatry and Behavioral Sciences, Medical University of South Carolina, Charleston, SC 29425, USA; cummingk@musc.edu

**Keywords:** cigarette, taxes, experimental tobacco marketplace, tobacco control

## Abstract

Taxes are a demonstrably effective method to suppress tobacco use. This study examined the effects of the tobacco parity (i.e., imposing taxes equally on all tobacco products) and the harm reduction (i.e., applying taxes in proportion to the products’ levels of harm) tax proposals on demand and substitution across products. A crowdsourced sample of cigarette smokers (*n* = 35) completed purchasing trials with increasing tax magnitudes across different tax tiers in the Experimental Tobacco Marketplace in a repeated-measures design. Products were placed in three tax tiers (high, medium, and no tax) according to each proposal’s goal. The results indicated that total nicotine (mg) purchased was not significantly different between the proposals, with higher taxes yielding lower demand. However, as taxes increased, the tobacco parity proposal decreased the purchasing of all tobacco products and increased the purchasing of medicinal nicotine (i.e., the no tax tier). Conversely, the harm reduction proposal resulted in greater purchases of electronic nicotine delivery systems and smokeless tobacco (i.e., the medium tax tier). These findings support tobacco taxation as a robust tool for suppressing purchasing and suggest that differential taxation in proportion to product risk would be an effective way to incentivize smokers to switch from smoked to unsmoked products. Further studies should investigate the unintended consequences of their implementation.

## 1. Introduction

While prior tobacco control efforts have focused primarily on conventional cigarettes, the tobacco marketplace’s growing complexity provides the tobacco user with numerous products. These products vary in harm and risks and may produce diverse outcomes, such as increased nicotine dependence [1], long-term use [2], and a reduced likelihood of quitting [3] due to poly-tobacco use.

There are several reasons why an individual may reduce tobacco use [4], with taxes being a widely used and demonstrably effective method [5,6]. Although prior studies have examined the impact of cigarette taxation on smoking, the study of taxes on other tobacco products (OTPs) is limited by retrospective analysis and related, in part, to the introduction of new products. For example, sales data suggest that introducing electronic nicotine delivery systems (ENDSs) taxes in Minnesota may have increased cigarette smoking prevalence and reduced quitting in the short term [7].

Substantially less is known about the effects of the simultaneous taxation of multiple tobacco products. The taxation of tobacco in the United States (US) is complicated by diverse taxing approaches across products and jurisdictions. First, tobacco taxes are levied at the local, state, and federal levels with considerable variability. For example, ENDSs are not federally taxed, while 28 states and DC have enacted taxes on ENDS products as of December 2020 [8]. Second, tobacco taxes differ by the specific product, such that taxes may be levied per unit (e.g., USD 2.00 per pack of cigarettes), ad valorem (e.g., 50% of the wholesale price), by weight (e.g., 1.00 USD/oz), or by volume (e.g., 0.10 USD/mL for E-liquids). Tobacco product manufacturers have exploited these complexities in the tax policy to avoid greater taxation. For example, many companies have re-labeled roll-your-own tobacco as pipe tobacco to be classified into lower tax brackets [9]. The complexity and ad hoc manner in which tobacco taxes are imposed is a barrier to public health efforts to discourage the use of tobacco products, especially products with well-established health risks. Further, the growing number of OTPs and paucity of studies on their taxation support the need for more research on how taxes affect purchasing behaviors.

Novel integrated tax proposals, such as the tobacco parity [10,11] and the harm reduction tax proposals [12,13,14,15], have been developed to reduce tobacco taxes’ complexity while promoting public health. These theoretical tax proposals are designed to achieve specific tobacco control end goals promoted by various key stakeholders (e.g., tobacco control experts, advocacy groups, and national and global health organizations). However, a consensus on the ultimate end-goal of tobacco control remains debatable, with some groups advocating for overall reductions in tobacco product use while others have focused on motivating users to switch to less harmful forms of tobacco [16].

The tobacco parity tax proposal would impose a higher tax equally on all nicotine-containing, non-therapeutic products, with no tax levied on medicinal nicotine/cessation products. The overarching goal is to encourage the cessation of all non-medicinal tobacco products and reduce opportunities for tax avoidance or evasion by tobacco users and companies. This proposal has been promulgated extensively by the World Health Organization’s Tobacco-Free Initiative [10] and the Campaign for Tobacco-Free Kids [11]. This proposal, however, does not take into consideration the harm differential that exists between different types of tobacco products and may inadvertently promote price-minimizing strategies for consumers, such as substituting cheaper but equally harmful combustible products, for example, generic cigarettes [17,18,19], cigars [20,21,22], and/or roll-your-own tobacco [19,23,24]. Further, this proposal is regressive, resulting in a disproportionate amount of the tax burden borne by low-socioeconomic status tobacco users who make up the majority of smoked tobacco product users.

The harm reduction tax proposal would levy taxes in proportion to the products’ levels of harm with the goal of transitioning tobacco users away from the most harmful products. The broadest definition of harm includes the effects on physical health and the likelihood of addiction (i.e., abuse liability). Proponents of tobacco harm reduction include several advocacy groups [12,13] and scientific experts [14,15]. Although novel products’ long-term effects are not clear, several of them have been documented to result in lower levels of harmful chemicals and biomarkers of harm compared to conventional cigarettes [25,26,27,28,29]. While a consensus exists that combustible tobacco produces the most significant harm [30,31,32], a product may be moved from one category to another as evidence accrues. Criticisms of this harm reduction tax proposal argue that cigarette smokers may not fully transition to less harmful products and the lower tax rates for these products may encourage non-smokers to use tobacco, increasing overall population-level health risks of dependence and harm [33,34,35,36].

Policies adopting either of these proposals would benefit from research that would provide evidence of their intended and unintended effects in different population groups (i.e., smokers, nonsmokers, adults, youth) before implementation. This study examined these tax proposals utilizing an innovative, validated methodology, the Experimental Tobacco Marketplace (ETM). The ETM is an experimental model of the “real-world” tobacco marketplace [37], in which tobacco users make virtual purchases among various tobacco products. In the ETM, the experimenter can control the price [38,39], flavor, and nicotine concentration [40,41] of each product. This methodology allows an understanding of the potential consequences of regulatory policies on consumers’ behaviors prior to policy implementation. One previous ETM study investigated the effect of cigarette taxes [42] on tobacco product purchasing while holding constant the prices of OTPs. However, no prior studies have explored the simultaneous taxation of multiple tobacco products. In the present study, we sought to do so while exploring the effects of a broad range of tax magnitudes, as one criticism of both tax proposals suggests that limited-magnitude taxes diminish the impact of these proposals. Thus, understanding how these tax proposals operate across a broad range of tax magnitudes may clarify their effects and identify the magnitudes that achieve or approximate the tax proposal’s goals.

The present experiment aimed to examine the effects of the tobacco parity and harm reduction tax proposals on demand for high tax products and the substitution of medium and no tax products among exclusive cigarette smokers. To accomplish this, we used a repeated measures design in the ETM. Of note, both proposals place conventional cigarettes in the high tax tier but vary in their placement of OTPs into the high, medium, and no tax tiers. In general, we hypothesized that both tax proposals would decrease the purchasing of high tax products and increase the purchasing of medium and no tax products. In addition, we hypothesized that the harm reduction proposal would result in a greater substitution of medium tax products. Furthermore, we hypothesized that the tobacco parity proposal would result in a greater substitution of no-tax products (i.e., nicotine replacement medications).

## 2. Materials and Methods

### 2.1. Participants

Participants were recruited into the study in December 2020 from Amazon Mechanical Turk (Mturk), a crowdsourcing platform to which employers post Human Intelligence Tasks (HITs) to be completed in exchange for monetary compensation [43]. Participants received USD 2.00 upon completing all questions. In addition, participants received a USD 3.00 bonus if their answers on the ETM instructional quiz containing 4 multiple-choice items demonstrated comprehension and attention.

Prior to enrollment, participants completed a screening questionnaire to determine their eligibility. Participants were eligible if they reported being 21 years of age or older, smoked 10 or more cigarettes per day, and indicated no regular use of alternative tobacco products. A total of 44 participants met the eligibility criteria and completed the study.

Consistent with previously published studies [44,45,46,47], the bonus was designed to increase attention to the survey. Only participants (*n* = 35) who mastered the quiz were included in the final analysis. See Appendix A Table A1 for a list of the participants’ usual cigarette brands. The participants provided electronic informed consent before beginning the study, which was approved by an Institutional Review Board at Virginia Polytechnic Institute and State University.

### 2.2. Procedure

The participants completed an online survey administered through Qualtrics survey software [48]. The median completion time of the survey was approximately 22 min. The survey included demographic questions, the Fagerstrom Test for Cigarette Dependence (FTCD) [49], the Questionnaire of Smoking Urges (QSU) [50], and the Perceived Health Risk scale (PHR) [51].

#### Experimental Tobacco Marketplace

Before entering the ETM, participants were given general instructions. They were provided with a list of products available in the ETM and asked to assume the following: a completely new purchasing scenario every time they had the opportunity to purchase items; they had no access to any other tobacco products other than those offered in the ETM; the products could not be given away or saved for longer than the next seven days; and the products were their preferred brand and flavor. Participants were asked to assume that they already had an e-cigarette device and a heating device or that they were available for free.

In the ETM, participants were exposed to two tax conditions, each consisting of six tax trials. In each trial, a hypothetical account balance was provided to purchase tobacco products for seven days.

Hypothetical account balance. The account balance was calculated by multiplying the self-reported average number of cigarettes smoked per day during the past month by the baseline unit price. This calculation mimics the income constraints faced by participants in real-world conditions [37,52].

Tax implementation. The order of the conditions was counterbalanced. Tobacco products were placed into three tax tiers: high, medium, and no tax, based on each proposal.

Tobacco parity tax proposal. The high tax tier included premium and generic cigarettes, 24 mg/mL of E-liquid, heat-not-burn stick, dip (finely ground or shredded, moistened smokeless tobacco product that users hold between their lower lip and their gum), and snus (a spit-less, smokeless tobacco product that users hold under their upper lip). The medium tax tier consisted of green tea cigarettes (nicotine-free) and 0 mg/mL of E-liquid. The no tax tier included 4 mg of gum and lozenges.

Harm reduction tax proposal. The high tax tier included premium, generic, and green tea cigarettes. The medium tax tier consisted of 24 mg/mL of E-liquid, 0 mg/mL of E-liquid heat-not-burn, dip, and snus. The no tax tier included gum and lozenges.

Purchase trials. Taxes increased across purchase trials according to different multiplicative factors (MF) in a logarithmic scale (high tax: baseline, 100%, 200%, 400%, 800%, and 1600%; medium tax: baseline, 12.5%, 25%, 50%, 100%, and 200%). The prices of the products in the no tax tier were held constant based on their baseline price.

To increase the resolution of the measurements, individual units were available in the ETM and not full packages. This method has been previously validated in prior studies [40,42,53,54,55,56]. E-Liquid was available in 0.1 mL bottles to approximately match the number of puffs per cigarette. Therefore, 1 cigarette was equivalent to 0.1 mL of E-liquid.

The baseline prices and units for each product were as follows: USD 0.38 per single premium cigarette (i.e., Marlboro, Camel, Newport), USD 0.28 per single generic cigarette (i.e., Eagle, Pyramid, or Rave), USD 0.24 per single green tea cigarette, USD 0.05 per 0.1 mL of E-liquid (24 mg/mL or 0 mg/mL), USD 0.40 per single heat-not-burn stick, USD 0.22 per single snus pouch, USD 0.10 per single dip pouch, and USD 0.37 per single piece of gum or lozenge. To determine the cigarette prices, first, the average of the price per carton of the three brands listed as generic and premium cigarettes from prices on shelbywholesale.com, a US distributor who ships to 42 out of 50 states, was calculated. Second, the average price was divided by 200, which is the number of cigarettes per carton. Green tea cigarettes and heat-not-burn prices per carton were retrieved from online stores to calculate the unit prices. Alternative tobacco product prices were defined by computing the median prices by product type using the Nielsen 2017 data [57]. Each product was displayed with the price along with an image and a brief description.

### 2.3. Data Analysis

#### 2.3.1. Sample Size Calculation

The total sample size for this study was *n* = 35. This sample size was calculated for a within-subject analysis of variance (ANOVA) using a medium effect size (*f* = 0.25), alpha of 0.05, and 80% power to test for differences in ETM product purchases between the two tax proposals.

#### 2.3.2. Participant Characteristics

Demographic characteristics and smoking-related assessments were described using the mean (standard deviation) and frequencies (percentages), where appropriate.

#### 2.3.3. Outcome Measures

Outcome measures of this study included the number of products and the total nicotine purchased in each trial. The total nicotine purchased in each trial was calculated by summing the quantity of nicotine purchased for each product available. The quantity of nicotine purchased for each product was calculated by multiplying the number of products purchased by the nicotine content of each product purchased (i.e., 10 mg per single premium or generic cigarette, 0 mg per single green tea cigarette, 2.4 mg per 0.1 mL of 24 mg/mL of E-liquid, 0 mg per 0.1 mL of 0 mg/mL of E-liquid, 15 mg per single heat-not-burn stick, 1 mg per single snus pouch, 1 mg per single dip pouch, 4 mg per single piece of gum or lozenge).

#### 2.3.4. Statistical Analysis

The total nicotine purchased was modeled using a repeated measures analysis of variance (ANOVA) with the trial and condition as the independent variables and a random effect associated with the participant. The total quantity of products purchased in each tax tier was modeled using a repeated measures ANOVA with interactions among the tax tier (high, medium, no tax), tax proposal, and trial, and a random effect associated with the participant. Post-hoc contrasts were performed to evaluate the purchasing and substitution of products in each tier. Two additional analyses were performed, one to compare premium and generic cigarette purchasing and another focusing on the purchasing of only e-cigarettes in the two tax proposals. All reported *p*-values have been adjusted for multiple testing (13 tests in total) using a false discovery rate (FDR) [58], and an adjusted *p*-value < 0.05 was considered significant. R software Version 4.0.5 was used for all data analyses [59].

## 3. Results

### 3.1. Demographics and Smoking-Related Assessments

Detailed sample characteristics regarding the demographics and tobacco-related measures are shown in Table 1. In general, the participants were 44.06 years of age and reported smoking 17.06 cigarettes per day, on average, which is indicative of a moderate degree of cigarette dependence.

### 3.2. Total Quantity of Nicotine Purchased

**Figure 1:**
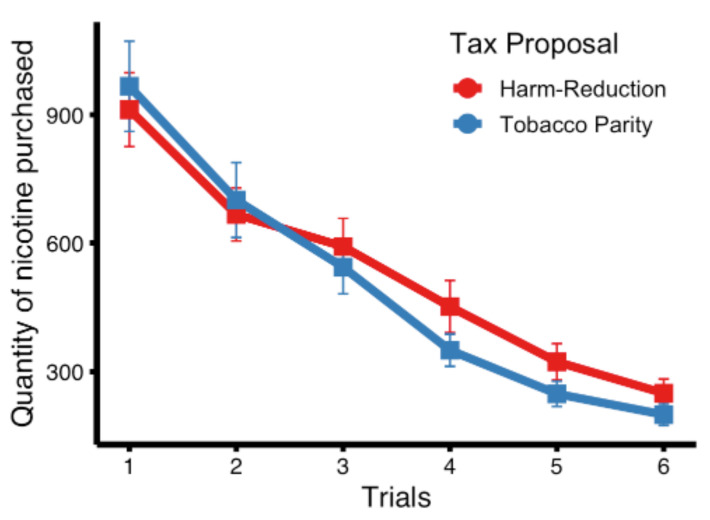
Total nicotine (mg) purchased in the tobacco parity and harm reduction tax proposals across trials.

The overall quantities of nicotine purchased from tobacco/NRT products between the tobacco parity and harm reduction tax proposals and across trials (Figure 1) were compared using a linear mixed-effects model. No statistically significant differences were observed between tax proposals (χ^2^ (1) = 2.093, *p* = 0.214) but the overall quantity of nicotine purchased decreased as the multiplicative tax factor increased (χ^2^ (1) = 501.510, *p* < 0.001).

### 3.3. Tax Proposals

**Figure 2:**
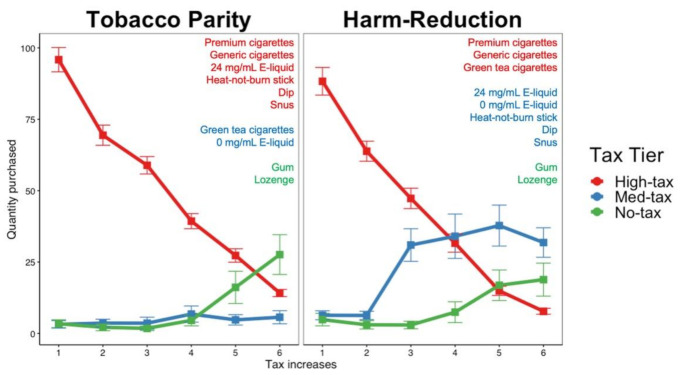
Total quantity of products purchased in the high, medium, and no tax tiers across trials in the tobacco parity and harm reduction tax proposals.

To test the hypothesis that both tax proposals would decrease the purchasing of high tax products and increase the purchasing of medium and no tax products as well as the differential substitution rates between proposals, we performed a repeated measures ANOVA. Figure 2 depicts the purchasing patterns in each tax tier for both tax proposals. Both the tobacco parity and harm reduction tax proposals decreased purchases from the highest tax tier as the tax rate increased (t(1214) = −10.684, *p* < 0.001; t(1214) = −10.888, *p* < 0.001, respectively). In addition, both tax proposals resulted in the substitution of no tax tier products (tobacco parity: t(1214) = 3.192, *p* = 0.003; harm reduction: t(1214) = 2.235, *p* = 0.042). However, the harm reduction tax proposal resulted in the substitution of the medium tax tier products (t(1214) = 4.333, *p* < 0.001) but no statistically significant substitution was observed in the tobacco parity tax proposals (t(1214) = 0.367, *p* = 0.772).

When comparing the purchasing patterns independently between the two tax proposals, significant differences were observed between the medium tax tiers (t(1214) = −2.808, *p* = 0.009), with the harm reduction tax proposal showing greater substitution than the tobacco parity tax proposal. No differences in purchasing were observed between the high tax products (t(1214) = 0.144, *p* = 0.885) and the no-tax products (t(1214) = 0.677, *p* = 0.589). That is, there was a similar pattern of decreasing purchases of high tax products and increasing purchases of no tax products as taxes increased across the two tax proposals.

Of note, the harm reduction tax proposal elicited more purchasing of E-liquid, with an increase of 7.75 units, on average (t(803) = 3.302, *p* = 0.003), or 0.775 mL. Independently of the tax proposal, generic cigarettes were purchased at higher quantities (4.8 cigarettes) than premium cigarettes (t(802) = 2.130, *p* = 0.435). See Appendix B Table A2 and Table A3 for a description of tobacco products purchased across trials in each tax proposal.

## 4. Discussion

The present study sought to investigate the effects of harm reduction and tobacco parity tax proposals on demand for high tax products and the substitution of medium and no tax products in exclusive cigarette smokers. Two main findings were observed: (1) no significant differences were identified in the total nicotine purchased from tobacco/nicotine replacement therapy (NRT) products across trials between the two tax proposals, (2) the tobacco parity and the harm reduction tax proposals decreased purchases from the high tax tier and increased substitution of no tax tier products as tax rates increased, but only the harm reduction tax proposal significantly increased purchases of the medium tax tier products. The findings are discussed below, with special consideration given to the tax proposals’ intended and unintended consequences on population health. Finally, we conclude by comparing the potential benefits of the tax proposals across these measures against the cost of these proposals in terms of their regressive nature (i.e., disproportionately affecting low-income individuals).

### 4.1. Total Nicotine Purchased from Tobacco/NRT Products

In terms of the intended and unintended consequences of taxes, total nicotine purchased may be informative as a proxy for changes in nicotine consumption that affect nicotine dependence and tobacco-related harm. One criticism of the harm reduction proposal is that many cigarette smokers may not fully transition to less harmful products and the lower tax rates for these products may encourage increased nicotine intake through poly-tobacco use, inadvertently increasing the risks of dependence and harm [33,34,35,36]. In the current study, the total nicotine purchased from tobacco/NRT products decreased as taxes increased but did not differ between the proposals. These data are generally consistent with prior work, indicating that the total nicotine purchased decreases as cigarette taxes increase [60]. However, these are the first data examining purchasing under naturalistic tax conditions in which the prices of multiple tobacco products increase simultaneously. These results strongly indicate that increases in cigarette taxes, independent of the structure of the tax proposal (i.e., level of tax imposed upon OTPs), reduce overall consumption of tobacco/NRT products among adults. These findings also support tobacco taxation as a robust tool for discouraging tobacco/NRT product purchasing and suggest that differential taxation of tobacco/NRT products in proportion to product risk would be an effective way to incentivize smokers to switch from smoked to low-risk, unsmoked types of tobacco.

### 4.2. Substitution between Products

Both tax proposals aim to improve population health by promoting the substitution of less harmful alternatives to cigarettes, although they differ as to which products are considered less harmful. The experimental investigation of the two tax proposals showed that their intended purposes may be achieved. Under the tobacco parity tax proposal, NRT was the preferred substitute since it was the only non-taxed option, suggesting that more cigarette smokers would transition to these products if high taxes were imposed on tobacco products. In contrast, the harm reduction proposal resulted in substitution with both medium tax and no tax products. Of note, ENDSs were the preferred alternative product in this tax proposal (See Appendix B Table A2). These results suggest that cigarette smokers would transition to less harmful products if high taxes were applied to more harmful products (i.e., combustible cigarettes).

The primary source of nicotine at lower tax trials remained combustible cigarettes in both tax proposals. Regardless of the trial, generic cigarettes were purchased in higher quantities than premium cigarettes, despite a majority of the sample’s usual brand being premium cigarettes. This observation could be consistent with price-minimizing strategies such as the substitution of cheaper but equally harmful products.

Taken together, these data indicate that the implementation of both tax proposals in the ETM produce their intended consequences (i.e., promoting the substitution of less harmful products at high-magnitude taxes of more harmful products), but additional studies need to be performed to fully elucidate any unintended consequences (i.e., promoting the substitution of equally harmful products at low-magnitude taxes). Importantly, the current data underscore concerns regarding the effective magnitudes that achieve or approximate each tax proposal’s goals. Further, recent data indicate that the presence of an illegal marketplace alters tobacco purchasing patterns in the ETM [61], with more restrictive conditions in the legal market increasing the likelihood of purchasing from the illegal market. Future research may wish to investigate whether the presence of an illegal marketplace would blunt the effects of the tax proposals observed here.

### 4.3. Limitations

A few limitations of this study must be acknowledged. First, the lack of racial and ethnic heterogeneity of the study sample prohibits further interpretation of the generality of the findings. Future studies should examine a more diverse sample to assess how tax proposals may interact with demographic and contextual factors. Second, because we used Mturk to recruit participants, only individuals previously approved by Amazon were able to complete the study. This might have introduced a sampling bias, which may limit generalizing results to more diverse samples than included in this study. Future studies are needed to examine these differential tax proposals to investigate the generality of our findings. Third, this study was restricted to participants who reside in the United States, and the effects of tax proposals on purchasing might be different in international samples. However, given that tax proposals are not specific to the United States, future studies could use the current methodology in an international sample. Fourth, the hypothetical nature of the ETM may represent a threat to external validity. However, previous studies using the ETM found that hypothetical purchasing and substitution are correlated with real-world use [62,63]. Lastly, for the purposes of experimental control, the current study required tax proposals to include three levels of tax (i.e., high, medium, and no tax). These tax levels and the products placed in each level may not be representative of how taxes might be implemented. For example, a country’s tax structure might include two or five tax levels and might place products in different tax levels than those examined here. Additionally, this study models a homogeneous tax environment, where one cannot drive across state lines, where taxes may be different, to subvert the goals of these proposals. The observed effects depend, in part, on tax policy at the federal level. Nevertheless, the current methodology provides a robust experimental model for examining how simultaneous taxation of multiple products at multiple tax levels affects purchasing. Future research could model the specifics of a particular country’s tax structure, for example, to get a more nuanced understanding of the effects of increasing tobacco taxes.

## 5. Conclusions

The current study makes several important contributions to the scientific literature. To our knowledge, this is the first research to examine the simultaneous taxation of multiple tobacco products. Prior studies utilizing both traditional and novel behavioral economic methods are limited by examining the effects of taxation on a single product (typically cigarettes or e-cigarettes). Thus, the current study extends prior research utilizing the ETM and represents a critical step forward for examining tobacco purchasing in a complex marketplace that includes multiple products and tax rates.

Our findings also suggest several potentially important implications for tobacco control policies that may provide key stakeholders with critical knowledge to inform their work. First, the data indicate that taxation remains a robust tool for controlling nicotine purchases. Second, our data seem to suggest that smokers will strongly defend their cigarette consumption (e.g., primarily purchase cigarettes) and only switch to primarily purchasing a substitute (e.g., OTPs) when exorbitantly high prices make cigarette smoking untenable. Third, the results indicate that the effects of the tax proposals were generally in line with their stated goals when high-magnitude taxes were implemented. Once cigarettes were priced out of smokers’ budgets, the differential OTP taxation across proposals pushed smokers toward different substitute products (i.e., low-taxed ENDSs for harm reduction and non-taxed NRTs for tobacco parity and harm reduction).

One criticism of the tax proposals was also supported by the current data. In particular, the effective magnitude required to accomplish the tax proposals’ goals is unrealistic given the very low-magnitude tax increases (e.g., USD 1.00) typically considered by policymakers. Overall, the differences between tax proposals emerged only after marked increases in cigarette taxes. This may favor the harm reduction taxation approach since it is more politically tenable to tax only the most dangerous forms of tobacco at a high level. Given that the intended consequences of both policies are only observed with very high cigarette taxes, concerns regarding the regressive nature of lower magnitude tax increases may be justified. Future research is needed to model the potential downstream health benefits (in terms of tobacco product consumption and the substitution of less harmful products) and inequities that might result from raising taxes on products disproportionately used by low-income individuals.

## Figures and Tables

**Table 1:** Demographic characteristics and tobacco-related measures’ means, standard deviations, and percentages.

	*n*	35
Demographics	Age (mean (*SD*))	44.06 (13.36)
	Gender = male (*n* (%))	17 (48.6)
	Race (*n* (%))	
	White	33 (91.4)
	Black or African American	1 (2.9)
	Asian	1 (2.9)
	Ethnicity = NOT Hispanic or Latino (*n* (%))	32 (91.4)
	Education (*n* (%))	
	High school (12 years)	4 (11.4)
	Some college (13–15 years)	15 (42.9)
	College (16 years)	11 (31.4)
	Graduate school (18 years)	5 (14.3)
Tobacco-related measures	Age of initiation (mean (*SD*))	17.57 (6.32)
	Cigarettes per day (mean (*SD*))	17.06 (8.98)
	FTCD (mean (*SD*))	5.60 (1.94)
	QSU cigarettes (mean (*SD*))	43.91 (16.63)
	PHR cigarettes (mean (*SD*))	70.23 (20.25)
	Willingness to try OTPs (%)	30 (85.71)

Note. FTCD: Fagerstrom Test for Cigarette Dependence; QSU: Questionnaire of Smoking Urges; PHR: Perceived Health Risk scale; OTPs: Other Tobacco Products.

## Data Availability

Readers are encouraged to email wkbickel@vtc.vt.edu to obtain more data for this study.

## Appendix A

**Table A1:** Participants’ usual cigarette brands.

Brand	*n* of Users
Basic	1
Camel	4
Eagle	1
Marlboro	18
Natural American Spirit	1
Pall Mall	2
USA	1
Winston	1
Other	6

## Appendix B

**Table A2:** Group mean and standard deviation of purchased tobacco products across trials in the tobacco parity tax proposal.

Tier	Tobacco Product	Market Price	MF 1	MF 2	MF 3	MF 4	MF 5
High tax	Premium cigarette	47.60 (54.91)	23.60 (32.86)	21.06 (38.20)	11.00 (20.52)	3.03 (6.20)	1.71 (3.20)
	Generic cigarette	46.86 (72.09)	44.80 (57.20)	29.94 (33.11)	19.37 (19.77)	12.09 (17.55)	5.23 (10.26)
	E-Liquid 24 mg/mL	0.26 (0.92)	0.23 (0.55)	6.91 (27.55)	8.09 (34.48)	11.57 (33.94)	6.80 (17.65)
	Heat-not-burn	0.46 (1.22)	0.49 (1.22)	0.66 (1.86)	0.54 (1.46)	0.29 (0.99)	0.20 (0.58)
	Snus	0.49 (2.06)	0.17 (0.51)	0.11 (0.40)	0.14 (0.43)	0.11 (0.40)	0.09 (0.37)
	Dip	0.23 (0.88)	0.14 (0.43)	0.20 (0.58)	0.17 (0.51)	0.20 (0.76)	0.11 (0.40)
Medium tax	Green tea Cigarette	2.26 (10.22)	3.00 (10.84)	3.29 (17.72)	6.00 (23.87)	3.71 (14.36)	2.46 (9.83)
	E-Liquid 0 mg/mL	0.91 (4.73)	0.60 (2.72)	0.29 (0.99)	0.77 (3.40)	1.03 (5.24)	3.20 (16.93)
No tax	Gum/Lozenge	3.40 (8.09)	2.14 (7.10)	1.77 (4.56)	4.57 (11.48)	16.11 (33.36)	27.60 (41.22)

**Table A3:** Group mean and standard deviation of purchased tobacco products across trials in the harm reduction tax proposal.

Tier	Tobacco Product	Market Price	MF 1	MF 2	MF 3	MF 4	MF 5
High tax	Premium cigarette	53.49 (54.68)	32.89 (40.46)	19.23 (38.29)	8.14 (14.54)	6.34 (14.58)	1.80 (3.50)
	Generic cigarette	34.03 (51.99)	30.74 (39.38)	27.80 (30.59)	23.14 (34.17)	8.03 (12.97)	5.80 (11.10)
	Green tea Cigarette	0.80 (3.71)	0.20 (0.47)	0.26 (0.56)	0.31 (0.93)	0.54 (2.54)	0.14 (0.55)
Medium tax	E-Liquid 24 mg/mL	3.51 (17.92)	4.94 (19.56)	18.89 (59.35)	31.46 (104.03)	35.37 (95.82)	28.91 (68.58)
	Heat-not-burn	0.57 (1.74)	0.43 (1.04)	4.29 (15.51)	2.23 (11.14)	1.77 (8.27)	1.86 (6.00)
	Snus	0.20 (0.53)	0.31 (0.96)	0.26 (0.89)	0.14 (0.49)	0.09 (0.28)	0.23 (0.73)
	Dip	0.20 (0.53)	0.17 (0.51)	0.14 (0.43)	0.11 (0.40)	0.11 (0.40)	0.49 (1.87)
	E-Liquid 0 mg/mL	1.86 (8.50)	0.43 (1.31)	7.37 (34.69)	0.09 (0.28)	0.43 (2.03)	0.34 (1.08)
No tax	Gum/Lozenge	4.83 (12.99)	3.00 (8.65)	2.94 (7.84)	7.43 (21.58)	16.86 (31.61)	18.83 (34.23)

## References

[1] Sung H.-Y., Wang Y., Yao T., Lightwood J., Max W. (2018). Polytobacco Use and Nicotine Dependence Symptoms Among US Adults, 2012–2014. Nicotine Tob. Res..

[2] National Center for Chronic Disease Prevention (1994). Preventing Tobacco Use among Young People: A Report of the Surgeon General.

[3] Mantey D.S., Chido-Amajuoyi O.G., Omega-Njemnobi O., Montgomery L. (2021). Cigarette Smoking Frequency, Quantity, Dependence, and Quit Intentions during Adolescence: Comparison of Menthol and Non-Menthol Smokers (National Youth Tobacco Survey 2017–2020). Addict. Behav..

[4] Yong H.-H., Borland R., Cummings K.M., Gravely S., Thrasher J.F., McNeill A., Hitchman S., Greenhalgh E., Thompson M.E., Fong G.T. (2019). Reasons for Regular Vaping and for Its Discontinuation among Smokers and Recent Ex-Smokers: Findings from the 2016 ITC Four Country Smoking and Vaping Survey. Addiction.

[5] Chaloupka F.J., Yurekli A., Fong G.T. (2012). Tobacco Taxes as a Tobacco Control Strategy. Tob. Control.

[6] Chaloupka F., Warner K.E., Culyer A.J., Newhouse J.P. (2000). The economics of smoking. Handbook of Health Economics.

[7] Saffer H., Dench D.L., Grossman M., Dave D.M. (2019). E-Cigarettes and Adult Smoking: Evidence from Minnesota.

[8] Boonn A. (2020). State Excise Tax Rating for Non-Cigarette Tobacco Products.

[9] Apuzzo M. (2009). Tobacco Execs Quickly Find Tax Loophole. San Diego Union-Tribune.

[10] World Health Organization (2010). WHO Technical Manual on Tobacco Tax Administration.

[11] Campaign for Tobacco Free Kids (2017). Creating Federal Tax Equity among All Tobacco Products Would Increase Federal Revenues & Promote Public Health.

[12] CASAA’s Mission. https://casaa.org/mission/.

[13] Boesen U. (2020). Taxing Nicotine Products: A Primer.

[14] Smith T.T., Hatsukami D.K., Benowitz N.L., Colby S.M., McClernon F.J., Strasser A.A., Tidey J.W., White C.M., Donny E.C. (2018). Whether to Push or Pull? Nicotine Reduction and Non-Combusted Alternatives—Two Strategies for Reducing Smoking and Improving Public Health. Prev. Med..

[15] Zeller M., Hatsukami D. (2009). Strategic Dialogue on Tobacco Harm Reduction Group the Strategic Dialogue on Tobacco Harm Reduction: A Vision and Blueprint for Action in the US. Tob. Control.

[16] McDaniel P.A., Smith E.A., Malone R.E. (2016). The Tobacco Endgame: A Qualitative Review and Synthesis. Tob. Control.

[17] Wang X., Xu X., Tynan M.A., Gerzoff R.B., Caraballo R.S., Promoff G.R. (2017). Tax Avoidance and Evasion: Cigarette Purchases from Indian Reservations Among US Adult Smokers, 2010–2011. Public Health Rep..

[18] DeCicca P., Kenkel D., Liu F. (2013). Who Pays Cigarette Taxes? The Impact of Consumer Price Search. Rev. Econ. Stat..

[19] Licht A.S., Hyland A.J., O’Connor R.J., Chaloupka F.J., Borland R., Fong G.T., Nargis N., Cummings K.M. (2011). Socio-Economic Variation in Price Minimizing Behaviors: Findings from the International Tobacco Control (ITC) Four Country Survey. Int. J. Environ. Res. Public Health.

[20] Boonn A. (2020). How to Make State Cigar Tax Rates Fair and Effective.

[21] Government Accountability Office (2011). Illicit Tobacco: Various Schemes Are Used to Evade Taxes and Fees.

[22] Government Accountability Office (2012). Tobacco Taxes: Large Disparities in Rates for Smoking Products Trigger Significant Market Shifts to Avoid Higher Taxes.

[23] Choi K., Hennrikus D., Forster J., St Claire A.W. (2012). Use of Price-Minimizing Strategies by Smokers and Their Effects on Subsequent Smoking Behaviors. Nicotine Tob. Res..

[24] Boonn A. (2020). The Problem with Roll-Your-Own (RYO) and Other Smoking Tobacco.

[25] Keith R.J., Fetterman J.L., Orimoloye O.A., Dardari Z., Lorkiewicz P.K., Hamburg N.M., DeFilippis A.P., Blaha M.J., Bhatnagar A. (2020). Characterization of Volatile Organic Compound Metabolites in Cigarette Smokers, Electronic Nicotine Device Users, Dual Users, and Nonusers of Tobacco. Nicotine Tob. Res..

[26] Stratton K., Kwan L.Y., Eaton D.L., National Academies of Sciences, Engineering and Medicine (2018). Public Health Consequences of E-Cigarettes.

[27] Shahab L., Goniewicz M.L., Blount B.C., Brown J., McNeill A., Alwis K.U., Feng J., Wang L., West R. (2017). Nicotine, Carcinogen, and Toxin Exposure in Long-Term E-Cigarette and Nicotine Replacement Therapy Users: A Cross-Sectional Study. Ann. Intern. Med..

[28] Stepanov I., Jensen J., Hatsukami D., Hecht S.S. (2008). New and Traditional Smokeless Tobacco: Comparison of Toxicant and Carcinogen Levels. Nicotine Tob. Res..

[29] Xia B., Blount B.C., Guillot T., Brosius C., Li Y., Van Bemmel D.M., Kimmel H.L., Chang C.M., Borek N., Edwards K.C. (2020). Tobacco-Specific Nitrosamines (NNAL, NNN, NAT, and NAB) Exposures in the US Population Assessment of Tobacco and Health (PATH) Study Wave 1 (2013–2014). Nicotine Tob. Res..

[30] Holman M.R. FDA Regulating Tobacco Products along a Continuum of Risk. Proceedings of the Tobacco Science Research Conference.

[31] Office of the Commissioner FDA Announces Comprehensive Regulatory Plan to Shift Trajectory of Tobacco-Related Disease, Death. https://www.fda.gov/news-events/press-announcements/fda-announces-comprehensive-regulatory-plan-shift-trajectory-tobacco-related-disease-death.

[32] Nutt D.J., Phillips L.D., Balfour D., Curran H.V., Dockrell M., Foulds J., Fagerstrom K., Letlape K., Milton A., Polosa R. (2014). Estimating the Harms of Nicotine-Containing Products Using the MCDA Approach. Eur. Addict. Res..

[33] Tomar S.L., Alpert H.R., Connolly G.N. (2010). Patterns of Dual Use of Cigarettes and Smokeless Tobacco among US Males: Findings from National Surveys. Tob. Control.

[34] Cirino P.T., Chin C.E., Sevcik R.A., Wolf M., Lovett M., Morris R.D. (2002). Measuring Socioeconomic Status: Reliability and Preliminary Validity for Different Approaches. Assessment.

[35] Siahpush M., Yong H.-H., Borland R., Reid J.L., Hammond D. (2009). Smokers with Financial Stress Are More Likely to Want to Quit but Less Likely to Try or Succeed: Findings from the International Tobacco Control (ITC) Four Country Survey. Addiction.

[36] Siahpush M., Farazi P.A., Maloney S.I., Dinkel D., Nguyen M.N., Singh G.K. (2018). Socioeconomic Status and Cigarette Expenditure among US Households: Results from 2010 to 2015 Consumer Expenditure Survey. BMJ Open.

[37] Bickel W.K., Pope D.A., Kaplan B.A., DeHart W.B., Koffarnus M.N., Stein J.S. (2018). Electronic Cigarette Substitution in the Experimental Tobacco Marketplace: A Review. Prev. Med..

[38] Quisenberry A.J., Koffarnus M.N., Hatz L.E., Epstein L.H., Bickel W.K. (2016). The Experimental Tobacco Marketplace I: Substitutability as a Function of the Price of Conventional Cigarettes. Nicotine Tob. Res..

[39] Quisenberry A., Koffarnus M.N., Bianco A., Perry E., Bickel W.K. (2017). The Experimental Tobacco Marketplace II: Substitutability in Dual E-Cigarette and Cigarette Users. Drug Alcohol Depend..

[40] Pope D.A., Poe L., Stein J.S., Kaplan B.A., Heckman B.W., Epstein L.H., Bickel W.K. (2019). Experimental Tobacco Marketplace: Substitutability of E-Cigarette Liquid for Cigarettes as a Function of Nicotine Strength. Tob. Control.

[41] Kaplan B.A., Pope D.A., Dehart W.B. (2019). Estimating Uptake for Reduced-Nicotine Cigarettes Using Behavioral Economics. Tob. Regul. Sci..

[42] Pope D.A., Poe L., Stein J.S., Kaplan B.A., DeHart W.B., Mellis A.M., Heckman B.W., Epstein L.H., Chaloupka F.J., Bickel W.K. (2019). The Experimental Tobacco Marketplace: Demand and Substitutability as a Function of Cigarette Taxes and E-Liquid Subsidies. Nicotine Tob. Res..

[43] Strickland J.C., Stoops W.W. (2019). The Use of Crowdsourcing in Addiction Science Research: Amazon Mechanical Turk. Exp. Clin. Psychopharmacol..

[44] Craft W.H., Tegge A.N., Bickel W.K. (2020). Episodic Future Thinking Reduces Chronic Pain Severity: A Proof of Concept Study. Drug Alcohol Depend..

[45] Athamneh L.N., Stein M.D., Lin E.H., Stein J.S., Mellis A.M., Gatchalian K.M., Epstein L.H., Bickel W.K. (2020). Setting a Goal Could Help You Control: Comparing the Effect of Health Goal versus General Episodic Future Thinking on Health Behaviors among Cigarette Smokers and Obese Individuals. Exp. Clin. Psychopharmacol..

[46] Stein J.S., Tegge A.N., Turner J.K., Bickel W.K. (2018). Episodic Future Thinking Reduces Delay Discounting and Cigarette Demand: An Investigation of the Good-Subject Effect. J. Behav. Med..

[47] Sze Y.Y., Stein J.S., Bickel W.K., Paluch R.A., Epstein L.H. (2017). Bleak Present, Bright Future: Online Episodic Future Thinking, Scarcity, Delay Discounting, and Food Demand. Clin. Psychol. Sci..

[48] Qualtrics (2021). Qualtrics Survey Software.

[49] Fagerstrom K. (2012). Determinants of Tobacco Use and Renaming the FTND to the Fagerstrom Test for Cigarette Dependence. Nicotine Tob. Res..

[50] Cox L.S., Tiffany S.T., Christen A.G. (2001). Evaluation of the Brief Questionnaire of Smoking Urges (QSU-Brief) in Laboratory and Clinical Settings. Nicotine Tob. Res..

[51] Mooney M.E., Leventhal A.M., Hatsukami D.K. (2006). Attitudes and Knowledge about Nicotine and Nicotine Replacement Therapy. Nicotine Tob. Res..

[52] Koffarnus M.N., Wilson A.G., Bickel W.K. (2015). Effects of Experimental Income on Demand for Potentially Real Cigarettes. Nicotine Tob. Res..

[53] DeHart W.B., Kaplan B.A., Pope D.A., Mellis A.M., Bickel W.K. (2019). The Experimental Tobacco Marketplace: Narrative Influence on Electronic Cigarette Substitution. Exp. Clin. Psychopharmacol..

[54] DeHart W.B., Mellis A.M., Kaplan B.A., Pope D.A., Bickel W.K. (2019). The Experimental Tobacco Marketplace: Narratives Engage Cognitive Biases to Increase Electronic Cigarette Substitution. Drug Alcohol Depend..

[55] Kaplan B.A., Koffarnus M.N., Franck C.T., Bickel W.K. (2020). Effects of Reduced-Nicotine Cigarettes Across Regulatory Environments in the Experimental Tobacco Marketplace: A Randomized Trial. Nicotine Tob. Res..

[56] Freitas-Lemos R., Stein J.S., Pope D.A., Brown J., Feinstein M., Stamborski K.M., Tegge A.N., Heckman B.W., Bickel W.K. (2021). E-Liquid Purchase as a Function of Workplace Restriction in the Experimental Tobacco Marketplace. Exp. Clin. Psychopharmacol..

[57] Retail Analytics. https://www.nielsen.com/apac/en/solutions/measurement/retail-analytics/.

[58] Benjamini Y., Hochberg Y. (1995). Controlling the False Discovery Rate: A Practical and Powerful Approach to Multiple Testing. J. R. Stat. Soc. Ser. B (Methodol.).

[59] R Core Team (2018). R: A Language and Environment for Statistical Computing.

[60] Quisenberry A.J., Koffarnus M.N., Epstein L.H., Bickel W.K. (2017). The Experimental Tobacco Marketplace II: Substitutability and Sex Effects in Dual Electronic Cigarette and Conventional Cigarette Users. Drug Alcohol Depend..

[61] Freitas-Lemos R., Stein J.S., Tegge A.N., Kaplan B.A., Heckman B.W., Cummings K.M., Bickel W.K. (2021). The Illegal Experimental Tobacco Marketplace I: Effects of VapingProduct Bans. Nicotine Tob. Res..

[62] Wilson A.G., Franck C.T., Koffarnus M.N., Bickel W.K. (2016). Behavioral Economics of Cigarette Purchase Tasks: Within-Subject Comparison of Real, Potentially Real, and Hypothetical Cigarettes. Nicotine Tob. Res..

[63] Heckman B.W., Cummings K.M., Hirsch A.A., Quisenberry A.J., Borland R., O’Connor R.J., Fong G.T., Bickel W.K. (2017). A Novel Method for Evaluating the Acceptability of Substitutes for Cigarettes: The Experimental Tobacco Marketplace. Tob. Regul. Sci..

